# Immunoproteomic identification of anti-C9 autoimmune antibody in patients with seronegative obstetric antiphospholipid syndrome

**DOI:** 10.1371/journal.pone.0198472

**Published:** 2018-06-12

**Authors:** Yoshimitsu Kuwabara, Akira Katayama, Sachiko Kurihara, Hideo Orimo, Toshiyuki Takeshita

**Affiliations:** 1 Department of Obstetrics and Gynecology, Nippon Medical School, Bunkyo-ku, Tokyo, Japan; 2 Department of Molecular Biology and Biochemistry, Nippon Medical School, Bunkyo-ku, Tokyo, Japan; CHA University, REPUBLIC OF KOREA

## Abstract

Immunoproteomic analysis was performed to identify unknown, pathology-related molecules in patients with seronegative (SN) obstetric antiphospholipid syndrome (APS) who clinically satisfied the diagnostic criteria for APS, but not the serological criteria. We collected peripheral blood from 13 SN-APS outpatients with known thrombotic predisposition, 13 with no known thrombotic predisposition, and four multiparous women with no history of miscarriage (control). Plasma proteins from volunteers were purified and used as plasma protein antigens. Two-dimensional immunoblotting was performed using pooled control or SN-APS serum samples as the primary antibodies. Mass spectrometry of reactive spots specific to SN-APS serum led to the identification of complement molecule C9. Western blotting using commercial purified alkylated C9 was performed to detect autoantibodies. Examination of individual patient serum identified reactivity in one patient with, and in two patients without known thrombotic predisposition. This study suggests that SN-APS pathologies were associated with autoantibodies that react to specific C9 epitopes.

## Introduction

Recurrent pregnancy loss (RPL) is defined as the loss of two or more pregnancies, the causes of which are diverse and not completely understood. Antiphospholipid antibody syndrome (APS) is one of the most established causes of RPL; this condition is diagnosed according to the international consensus classification criteria [1]. Currently, anti-thrombotic therapy using a combination of aspirin and heparin during pregnancy is widely employed as a standard therapy for obstetric APS [2]. In addition, patients with non-criteria obstetric APS (NC-APS) who do not sufficiently meet the diagnostic criteria for obstetric-APS, and those with seronegative obstetric APS (SN-APS) who do not satisfy the serological criteria for obstetric-APS, have also been reported [3]. In these patients, the etiology of RPL is classified as unknown, and there is no established treatment policy for this condition. According to some observational and cohort studies, the prognosis of pregnancy in SN-APS and NC-APS is likely to be negatively affected by underestimation of these conditions, which leads to a judgment that treatment is not necessary [4]. Therefore, it is important to develop an improved therapeutic approach, based on the underlying pathophysiological mechanisms. Since high-dose immunoglobulin therapy for RPL associated with SN-APS has been reported to be effective [5], autoimmune mechanisms associated with specific antigens may be involved in these cases, as they are in APS.

Antiphospholipid antibodies do not react with phospholipids per se; rather, they detect plasma proteins such as beta2GP1 and prothrombin, which bind to phospholipids [6, 7]. Therefore, it is possible that a novel autoantibody against an unknown plasma protein may be involved in the pathophysiology of SN-APS. Immunoproteomics is an established technique that involves immunoblotting subsequent to two-dimensional electrophoresis (2-DE), and provides a useful approach for identifying novel disease-related molecules in various conditions, including autoimmune disease, cancer, and infection [8]. Recently, new autoantibodies related to the pathology of neuropsychiatric SLE [9], Hashimoto’s encephalitis [10], multiple sclerosis [11], and Crohn’s disease [8] have been identified using this technique. In the present study, we applied an immunoproteomic approach to identify novel, pathology-related molecules in patients with SN-APS.

## Materials and methods

### Clinical specimens

This investigation was conducted according to the principles expressed in the Declaration of Helsinki. Study protocols were approved by the medical ethics committee of Nippon Medical School Hospital. Written informed consent for study participation was obtained from all subjects. SN-APS was defined as RPL that met the clinical criteria for obstetric APS, i.e., 1) one or more unexplained deaths of a morphologically normal fetus at or beyond the 10th week of gestation, or 2) three or more unexplained consecutive spontaneous miscarriages before the 10th week of gestation, without maternal anatomical or hormonal abnormalities, and 3) negative results in serological evaluations of lupus anticoagulant, anti-cardiolipin IgG, anti-cardiolipin IgM, and anti-β2 glycoprotein I antibodies. To screen for known thrombophilic factors other than the serological items included in the international consensus classification criteria of APS, the presence or absence of anti-phosphatidylethanolamine (PE) antibody, protein S value, protein S activity, protein C value, protein C activity and factor XII value were determined. Positivity for the anti- PE antibody was defined as more than 95th percentile in titer of anti-PE antibody IgM kininogen (+) and/or anti-PE antibody IgG kininogen (+). Protein S deficiency was defined by the presence of < 65% plasma protein S and/or < 60% protein S activity; protein C deficiency was defined by the presence of < 70% plasma protein and or < 64% protein C activity; factor XII deficiency was defined by the presence of < 60% factor XII levels. Protein C deficiency was not observed in all specimens. We collected peripheral blood from SN-APS outpatients with a known thrombotic predisposition (n = 13), those with no known thrombotic predisposition (n = 13), and multiparous women with no history of miscarriage (n = 4). Blood samples were incubated for 20 min at room temperature and centrifuged for 15 min at 3,500 rpm; the supernatant was pipetted into polystyrene tubes and stored at -80°C until analysis. The characteristics of the subjects are summarized in Table 1.

**Table 1:** Sample characteristics and experimental results.

Sample characters							Results	
Sample no.	Age (years)	No. of fetal losses	Without thrombophilia	Positive for anti-PE antibody	Protein S deficiency	Factor XII deficiency	Positive for anti-C9 antibody	C9 (μg/ml)
1	41	8	**○**					67.18
2	43	3		**○**				52.54
3	30	2		**○**	**○**			52.54
4	30	3			**○**		◎	70.46
5	40	3		**○**	**○**	**○**		65.48
6	36	7		**○**				112.4
7	35	4	**○**					47.36
8	30	4	**○**					25.06
9	36	3	**○**					51.24
10	37	2	**○**					21.88
11	27	3		**○**				124.04
12	40	5	**○**					55.64
13	43	4	**○**					47.76
14	31	3			**○**			40.1
15*	41	3	**○**				◎	104.62
16*	31	4	**○**				◎	67.28
17*	30	3	**○**					53.64
18	36	2	**○**					40.8
19	39	6				**○**		34.12
20	39	3			**○**			29.44
21	37	3	**○**					44.98
22	38	3		**○**		**○**		45.98
23	40	3				**○**		45.18
24	38	3	**○**					36.8
25	42	4			**○**			94.76
26	33	3			**○**			56.42
Cont 1	32	0						84.42
Cont 2	30	0						58.22
Cont 3	38	0						32.92
Cont 4	36	0						52.34

Asterisk indicates pooled SN-APS serum sample.

### Plasma protein separation by 2-DE

Blood from six female volunteers was collected into anti-coagulant-containing Spitz tubes (BD-P100; Becton Dickinson). Plasma was purified using an albumin and IgG removal column (GE Healthcare) to obtain plasma protein antigens. 2-DE was performed using a Multiphor II horizontal electrophoresis system (Amersham Pharmacia, NJ, USA) according to the manufacturer’s instructions. One-dimensional isoelectric focusing (IEF) was performed on a precast 18-cm Immobiline dry strip (Amersham Pharmacia). Proteins (ca. 1 mg) for Coomassie Brilliant Blue (CBB) and silver nitrate staining (50 μg) were mixed with a re-hydration buffer containing 7.5 M urea, 2 M thiourea, 4% CHAPS, 2% dithiothreitol (DTT), 2% immobilized pH gradient (IPG) buffer (Amersham Biosciences), and a few grains of bromophenol blue (as the tracking dye) in a total volume of 330 μl. The mixtures were loaded onto the IPG strip. After re-hydration for 12 h, IEF was performed under the following conditions: (i) 500 V, 1 Vh; (ii) 3500 V, 3,000 Vh; and (iii) 3,500 V, 30,000 Vh. After IEF, the IPG strips were equilibrated with equilibration buffer I (containing 50 mM Tris–HCl pH 6.8, 6 M urea, 2% sodium dodecyl sulfate [SDS], 30% glycerol, 1% DTT) and buffer II (containing 50 mM Tris–HCl pH 6.8, 6 M urea, 2% SDS, 30% glycerol, 2.5% iodoacetamide, and a few grains of bromophenol blue). The strips were then subjected to gradient SDS-polyacrylamide gel electrophoresis (PAGE) in 12–14% polyacrylamide gels. SDS-PAGE was run at 12 mA for 1 h, 40 mA for 5 min, and finally at 40 mA for 2 h 40 min after removal of the strips. At the end of each run, the gels were stained with silver nitrate. The stained 2-DE gels were scanned on an ES-2200 instrument (EPSON, Nagano, Japan), and the data were analyzed using an ImageMaster 2D Elite system (Amersham Biosciences).

### Identification of immunoreactive spots specific to SN-APS serum

The separated plasma proteins on the 2-DE gels were electroblotted onto a polyvinylidene difluoride (PVDF) membrane (Fluor Trans, Japan Genetics, Tokyo, Japan) using a semi-dry blotting apparatus (Nova Blot, Amersham Pharmacia) at 0.8 mA/cm^2^ for 25 min. The blotting buffer consisted of 25 mM Tris–HCl pH 8.3, 192 mM glycine, 0.1% SDS, and 20% methanol. The membrane was blocked with PVDF Blocking Reagent For Can Get Signal (Toyobo, Osaka, Japan) for 1 h, and then incubated overnight with either SN-APS or control serum, which was diluted 1:200 in Can Get Signal Solution 1 (Toyobo, Osaka, Japan). To save the expense and time for screening, three SN-APS sera with no known thrombophilia were pooled in a SN-APS mix and subjected to immunoblotting. The control serum was pooled from four multiparous women with no history of miscarriage. The membrane was then incubated with sheep anti-human IgG horse radish peroxidase (HRP)-linked whole Ab (GE Healthcare) in Can Get Signal Solution 2 (Toyobo, Osaka, Japan) for 1 h. The bound conjugates were detected with enhanced chemiluminescence (ECL) Prime (GE Healthcare), and spots specific to the SN-APS mix were identified.

### In-gel enzyme digestion and mass spectrometry

Three protein spots specific to the SN-APS serum with similar molecular weights and isoelectric points were excised from the 2-DE gels, washed with deionized water and acetonitrile, and dried in a SpeedVac (Thermo Savant, NY, USA) for 30 min. Samples were pooled and reduced using 100 mM DTT in 100 mM ammonium bicarbonate for 30 min at 56°C prior to alkylation using 100 mM iodoacetamide in 100 mM ammonium bicarbonate for 30 min at 37°C in the dark. The gel-strips were then washed in 100 mM ammonium bicarbonate and acetonitrile, and vacuum-dried. The protein was digested overnight in 12.5 ng/μl proteomics-grade recombinant trypsin (Roche) in 10 mM Tris-Hcl (pH 8.8) at 37°C. Peptides were further extracted from the supernatant using 5% formic acid and 70% acetonitrile. The peptide extracts were vacuum-dried and resuspended in 20 μl 0.1% trifluoroacetic acid.

Peptides were subjected to a nano-flow liquid chromatographic separation on an advanced NanoLC system (Michrom BioResources Inc.) with a reverse-phase capillary high-performance liquid chromatography column (Zaplous α Pep-C18; 0.1 mm × 50 mm; AMR, Inc., Tokyo, Japan) and amaZon ETD ion trap (Bruker Daltonics) equipped with a CaptiveSpray NSI source (Bruker Daltonics). Database searching was conducted using the SwissProt database and Mascot software (version 2.5, Matrix science).

### Alkylation of C9

To subject the commercial purified human C9 (C3660; Sigma) to the same protein preparation processes used for 2-DE, C9 in 8 M urea was reduced with 28 mM DTT for 2 h, and subsequently alkylated in 41 mM iodoacetamide at 37°C for 2 h in the dark. The alkylated sample was diluted with SDS sample buffer (0.25 M Tris-HCl, pH 6.8, 2% SDS, 5% 2-mercaptoethanol, and 5% glycerol). To confirm the effect of urea-mediated denaturation on immunoreactivity, C9 dissolved in phosphate buffered saline was also alkylated under the same condition and diluted with SDS sample buffer.

### Western blot analysis

To detect C9 auto-antibodies in each serum sample, solubilized purified C9 (with or without alkylation) in SDS sample buffer was subjected to SDS-PAGE using 4–20% Mini-PROTEAN TGX precast gels (Bio-Rad), and electro-blotted onto a PVDF membrane using a Trans-Blot Turbo Transfer mini pack (Bio-Rad). The electrophoresed lanes containing C9 on the membrane were cut into strips based on the position of the molecular weight marker and the dye marker (pink band) (S1 Fig). Each membrane strip was blocked with PVDF Blocking Reagent for Can Get Signal (Toyobo, Osaka, Japan) for 1 h prior to overnight incubation with the SN-APS mixed sera, control mixed sera, or individual serum samples (diluted 1:500) in Can Get Signal Solution 1 (Toyobo, Osaka, Japan). Each membrane strip was then incubated with sheep anti-human IgG HRP-linked whole Ab (GE Healthcare) (1:10,000) diluted in Can Get Signal Solution 2 (Toyobo, Osaka, Japan) for 1 h. The bound conjugates were detected using ECL Prime (GE Healthcare) and a LAS-4000 luminescent image analyzer (Fuji Film).

### Enzyme-linked immunosorbent assay (ELISA)

C9 levels in serum samples were assayed using a human complement C9 ELISA kit (ab137972, MA, USA) according to the manufacturer’s instructions.

### Measurement of complement activity

Complement hemolytic activities of the anti-C9 antibody-positive samples with or without addition of purified C9 were evaluated using sheep red blood cells and the CH50 Seiken kit (Denka Seiken, Niigata, Japan) following the manufacturer’s instructions.

## Results

### Identification of plasma proteins reacting with SN-APS serum

**Fig 1:**
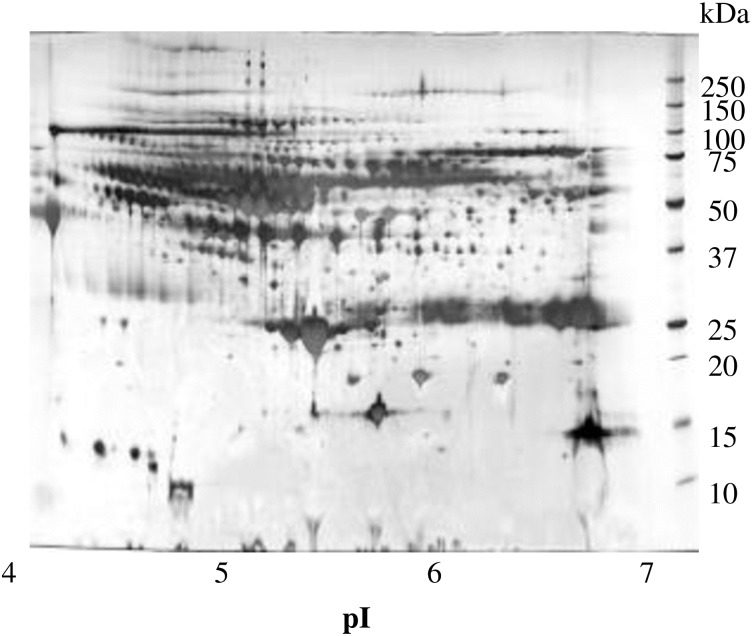
Separation of plasma proteins by 2-DE. Blood from six female volunteers was purified to provide plasma protein antigens, which were subjected to 2-DE and stained with silver nitrate.

Purified plasma proteins were separated using 2-DE to obtain protein spots (Fig 1).

**Fig 2:**
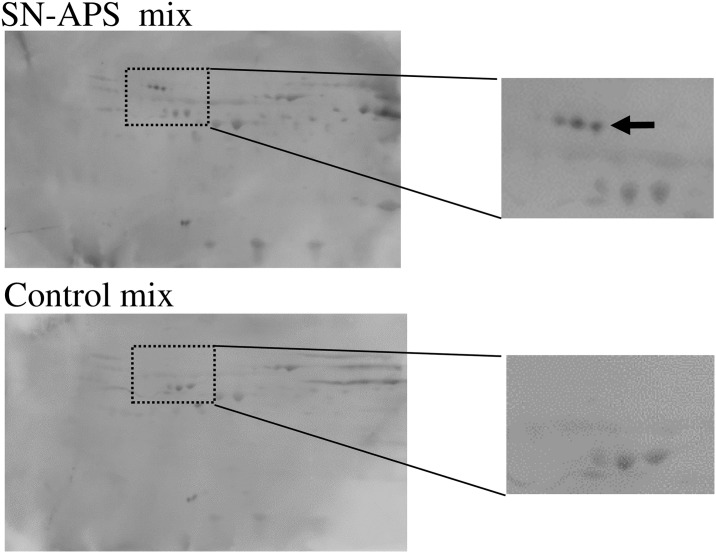
Identification of immunoreactive spots specific to SN-APS serum by 2-DE. Following plasma 2-DE, the membranes were incubated with pooled serum from either controls or SN-APS patients (numbers 15, 16, and 17), and immunoreactive spots specific to SN-APS serum were identified, as indicated by the arrow. Enlarged views are shown on the right-hand side.

Differential analysis of 2-DE membranes incubated with either pooled control serum or pooled serum from SN-APS patients revealed three unique SN-APS-specific protein spots with similar molecular weight and isoelectric point in the pool of SN-APS serum sample nos.15, 16, and 17 (Fig 2). Proteins extracted from these spots were pooled and subjected to mass spectrometry. The Mascot search showed that only the complement molecule C9 had high score, whereas the other candidate proteins did not match the molecular weight according to their positions in 2-DE analysis (S1 Table). The observed pI and molecular weight in 2-DE analysis were also similar to those reported previously for C9 [12]. Therefore, it appears that all three spots were C9, albeit with different modifications. The raw mass spectrometry data for identifying the reactive spots by LC-MS/MS spectra and Mascot search are shown in Supporting Information (S2 Fig).

### Establishment of a western blotting detection system for the anti-C9 antibody

**Fig 3:**
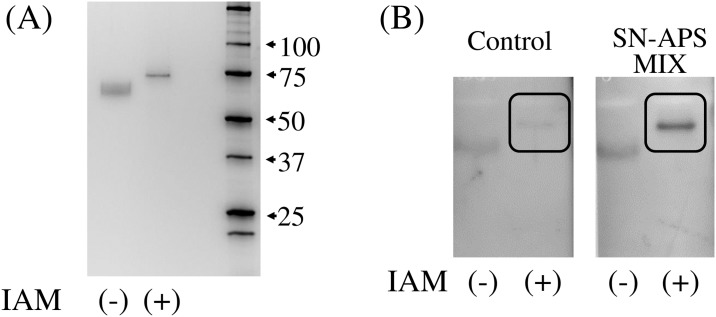
Anti-C9 antibody detection by western blotting using commercially available purified C9. Purified C9 (100 ng), with or without alkylation by iodoacetamide (IAM), was subjected to SDS-PAGE. (A) Alkylated C9 showed sharper band of higher molecular weight. (B) The band corresponding to purified alkylated C9 was clearly detected by pooled serum from SN-APS patients.

Contrary to the results of 2-DE immunoblotting, SDS-PAGE, followed by western blotting indicated that the pooled serum from SN-APS patients (numbers 15, 16, and 17) did not react with the commercially available purified C9 and electrophoresed as a single band on SDS-polyacrylamide gel. To eliminate the possibility that a protein preparation step during 2-DE had affected antigenicity, the purified C9 was subjected to alkylation by iodoacetamide (similar to the 2-DE preparation step). After the iodoacetamide treatment, purified C9 showed sharper bands in SDS-PAGE of higher molecular weight (Fig 3A). In the subsequent western blotting analysis, the C9 band was clearly detected by the pooled serum from SN-APS patients, allowing detection of the anti-C9 antibody (Fig 3B).

### Analysis of the anti-C9 antibody in individual serum samples

**Fig 4:**
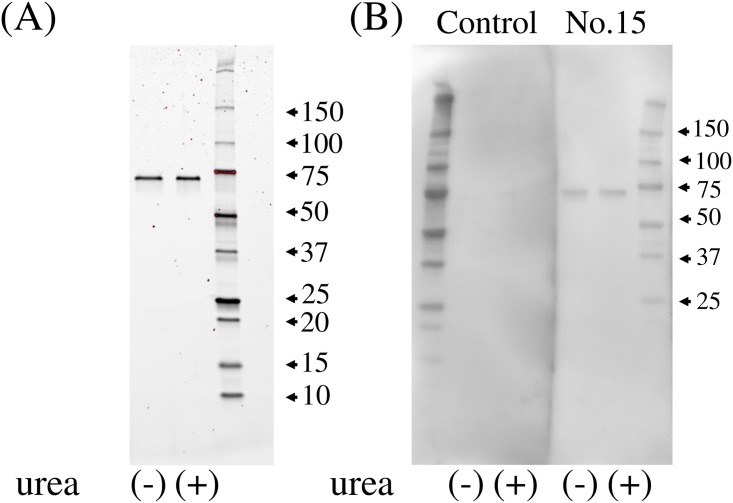
The effect of urea-mediated denaturation on immunoreactivity to alkylated C9. Purified alkylated C9 (100 ng), dissolved with or without 8M urea prior to IAM treatment, were subjected to SDS-PAGE (A). Serum sample no. 15 reacts with alkylated C9 regardless of urea denaturation (B).

Prior to conducting experiments for the detection of anti-C9 antibody in individual serum samples, we confirmed that serum sample no. 15, a constituent of the SN-APS mix, reacts with alkylated C9. Using this serum, we also evaluated whether generation of immunoreactivity to the C9 antigen by alkylation depends on urea-mediated denaturation. Purified C9, dissolved with or without 8M urea prior to IAM treatment, were subjected to SDS-PAGE (Fig 4A). Subsequent western blotting demonstrated that serum sample no. 15 reacts with alkylated C9 regardless of denaturation (Fig 4B). Based on these results, urea-denatured C9 was used as an antigen, and serum sample no.15 was used as a positive control in the following experiment.

**Fig 5:**
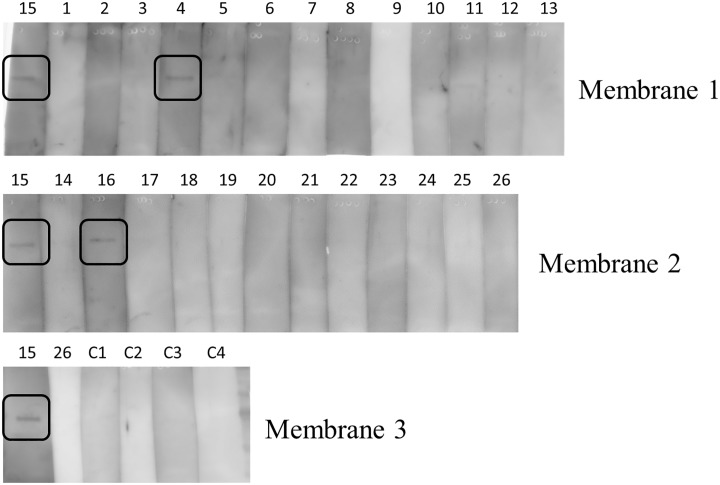
Analysis of the anti-C9 antibody in individual serum samples from SN-APS patients. Purified alkylated C9 (50 ng) was subjected to SDS-PAGE thrice, and blotted onto PVDF membranes (membrane 1, 2, and 3). Each membrane was cut into strips corresponding to the electrophoresed lanes and reacted with individual patient serum samples. As a positive control, serum no. 15 was included on a strip from each membrane. In the figure, the cut strips that were subjected to western blotting are arranged and shown in the order of loading on the original membrane. The lines separating the lanes were not generated by image processing, but are artifacts of the experimental procedure used to separate parts of the membrane for reaction with different serums. Anti-C9 antibody was detected in two of 13 SN-APS patients without known thrombophilia, one of 13 SN-APS patients with thrombophilia, and none of the four control samples.

To include all samples, SDS-PAGE of iodoacetamide-treated purified C9 was performed thrice and blotted onto PVDF membranes (membrane 1, 2, and 3). Each membrane was cut into strips corresponding to the electrophorated lanes and reacted with individual patient serum samples. As a positive control for correct protein blotting, serum sample no. 15 was incubated with strips from all membranes and shown as a band on the left end lane of each membrane. In S1 Fig, the separated strips were combined and shown together with the rest of membrane, including a part of protein marker to show that the appropriate part was cut out from the same membrane. As a result, anti-C9 antibody was detected in 2 of 13 specimens from SN-APS patients without known thrombophilia (no.15, No.16), and in 1 of 13 specimens of SN-APS patients with thrombophilia (No. 4); no band was detected in the four control samples (Fig 5). To investigate the relationship between the anti-C9 antibody and congenital C9 deficiency, we evaluated the C9 levels in each serum sample using ELISA, which confirmed that there was no significant association between anti-C9 antibody positivity and C9 level (Table 1). In addition, the CH50 activity of the anti-C9 antibody-positive samples was in the normal range, and showed no change after addition of purified C9 (no. 4: 28.3⇒29.0U/ml, no. 15: 33.9 ⇒ 34.9 U/ml, no. 16: 24.4 ⇒ 22.2 U/ml).

## Discussion

In this study, autoantibodies that specifically reacted with complement molecule C9 were detected in the serum of approximately 11% SN-APS patients. C9 acts as the final component of the complement cascade, forming a membrane attack complex (MAC) with C5b, C6, C7, and C8 in the final step of complement activation [13]. C9 polymerizes with itself to form poly-C9, which is a major component of the MAC [14]. The anti-C9 antibody detected in this study specifically recognized iodoacetamide-treated C9, without reacting with conventional purified C9, suggesting that this autoantibody may not react with free C9 circulating in the blood. We have also confirmed that the anti-C9 antibody recognizes iodoacetamide-treated C9 regardless of denaturation by urea. On alkylation, cleaved disulfide bonds generated by DTT treatment were maintained because of capping of the SH residue by iodoacetamide. Therefore, when iodoacetamide-treated C9 is used as an antigen, the anti-C9 antibody specifically recognizes an epitope generated by a structural change that is dependent on disulfide bond cleavage. Based on our current understanding of C9 structure, it may be possible to verify whether the anti-C9 antibody recognizes structurally altered C9 such as poly-C9 within the MAC in vivo.

Congenital C9 deficiency is one of the most frequent inherited diseases in Japan, and is reported to exist in approximately 0.1% of the Japanese population investigated in the present study [15]. Most people with C9 deficiency are asymptomatic and reports indicating any relationship between C9 deficiency and RPL are lacking. Because C9 levels were in the normal range in all SN-APS serum samples examined, the involvement of C9 deficiency in SN-APS pathology was not suggested. Furthermore, in combination with the CH50 activity measurements, the presence of anti-C9 antibody does not appear to contribute to C9 deficiency either quantitatively or functionally.

Complement activity is strictly regulated by the expression and localization of complement regulatory factors at the feto-maternal interface, and plays an important role in the establishment and maintenance of pregnancy. Observations from immunohistochemical staining of clinical specimens and rodent models indicate that excessive or misdirected complement activation increases the risk of pregnancy loss [16]. Therefore, it is interesting that the present study detected an antibody against a complement factor in patients with RPL of unknown etiology. Investigation of a mouse abortion model that employs an antiphospholipid antibody showed that C5b (derived from the classical complement pathway) was directly involved in the induction of miscarriage, whereas subsequent MAC formation was not essential [17]. In contrast, immunohistochemical analyses have identified a wide distribution of MAC around the deciduous blood vessels of patients with RPL or hypertensive disorders of pregnancy [17, 18]. These findings suggest that the anti-C9 antibody detected in patients with SN-APS may not be directly involved in the mechanism underlying miscarriage, but may instead reflect excessive or misdirected complement activation caused by the loss of an earlier pregnancy. In this regard, the anti-coagulants that are administered as the standard therapy for APS may also be effective in anti-C9 antibody-positive SN-APS patients because heparin suppressed complement activation in antiphospholipid-antibody treated mice and assisted in preventing pregnancy complication [19]. In further refractory cases, the use of eculizumab might provide an additional option. This antibody acts against C5 and is currently used to treat paroxysmal nocturnal hemoglobinuria, which is characterized by chronic C5 activation.

The presence of the anti-C9 antibody can be correlated with RPL associated with complement activation, with or without the involvement of antiphospholipid antibodies. To clarify this point, it is important to perform larger studies using serum from patients with RPL, including APS, in the future. It is also important to investigate the presence or absence of anti-prothrombin antibody, which has recently been suggested to be strongly associated with APS pathophysiology. Further investigation of the relationship between the presence of anti-C9 antibody and both serological and clinical features of RPL patients may lead to the development of a novel clinical marker that can be used to inform treatment decisions for patients with refractory RPL.

## Supporting information

S1 Fig Combined separated strips shown together with the rest of membrane including a part of protein marker. The electrophoresed lanes containing C9 on the membrane were cut into strips based on the position of the molecular weight marker and the dye marker (pink band).
(PDF)

S2 Fig Protein identification of reactive spot by LC-MS/MS spectra and Mascot search. Among the identified candidate proteins, the C9 protein shows remarkably high score (2785) as compared with the second candidate protein (Alpha-1B-glycoprotein, gene A1BG, score 300). Representative MS/MS spectrums of particular peptides of C9 protein are shown. The red peaks indicate matched b-ion and y-ion series. The sequences of precursor ions, ([M + 2H]^2+^, a:733.296, b:1395.453, c:728.271), were analyzed by MS/MS to be DVVLTTTFVDDIK (a), GTVIDVTDFVNWASSINDAPVLISQK (b), and AIEDYINEFSVR (c).
(PDF)

S1 Table Results of Mascot search. The Mascot search showed that only the complement molecule C9 had high score, whereas the other candidate proteins did not match the molecular weight according to their positions in 2-DE analysis.
(PDF)
