# Supplementary figures and images for: Immunoproteomic identification of anti-C9 autoimmune antibody in patients with seronegative obstetric antiphospholipid syndrome
Source: PLoS One. 2018 Jun 12;13(6):e0198472. doi: 10.1371/journal.pone.0198472 (PMC5997311; doi:10.1371/journal.pone.0198472)

S1 Fig

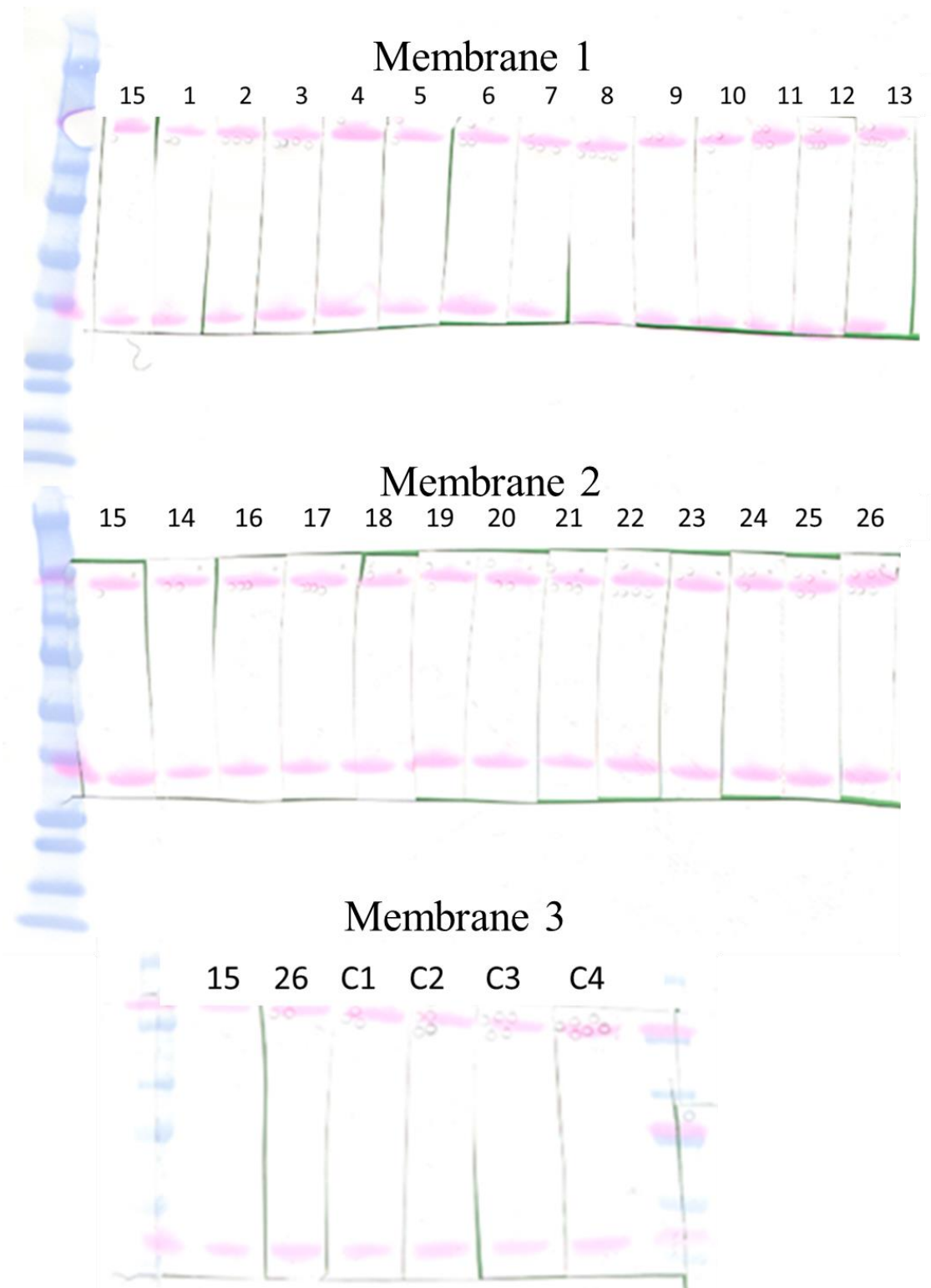

Supplement: S1 Fig — The electrophoresed lanes containing C9 on the membrane were cut into strips based on the position of the molecular weight marker and the dye marker (pink band). (PDF) [file pone.0198472.s001.pdf]

# S2 Fig

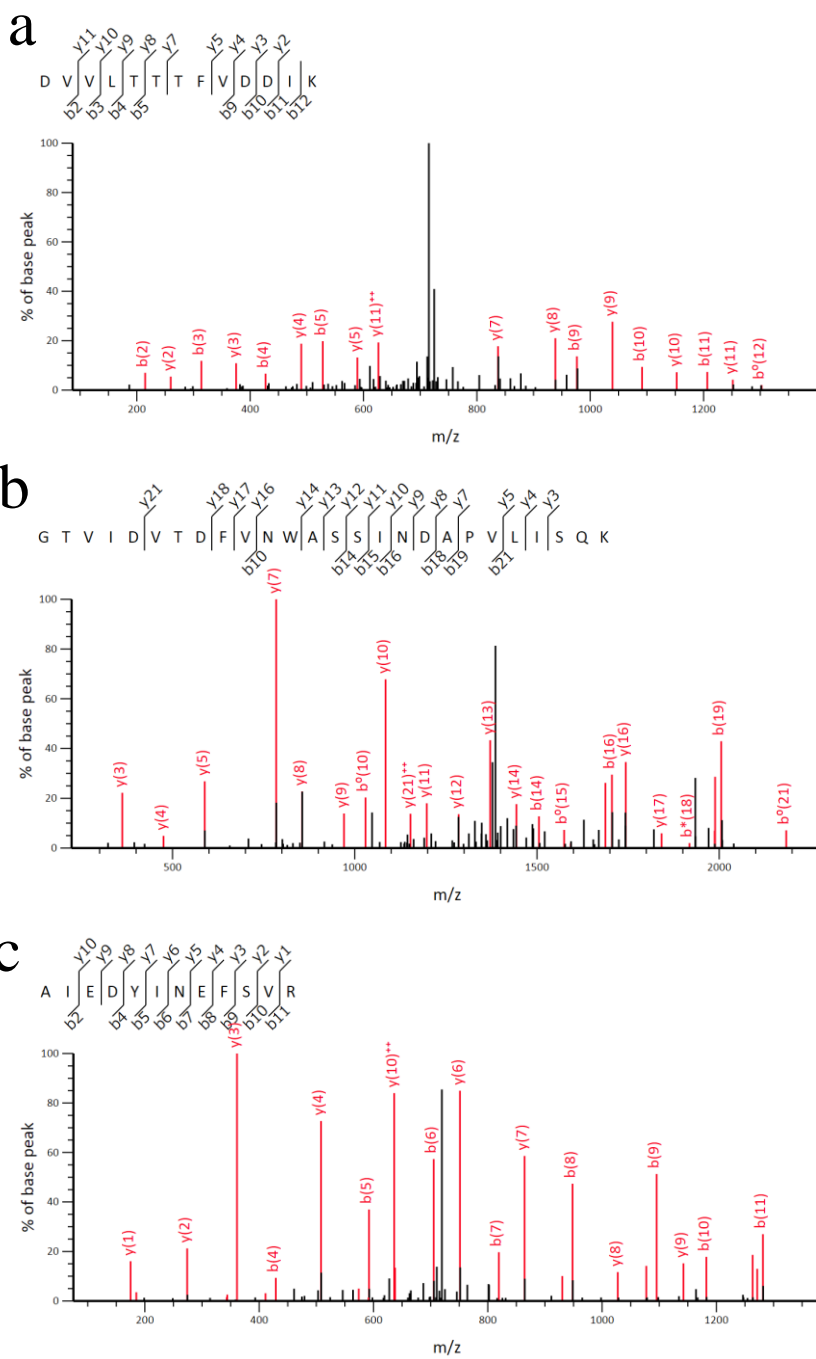

Supplement: S2 Fig — Among the identified candidate proteins, the C9 protein shows remarkably high score (2785) as compared with the second candidate protein (Alpha-1B-glycoprotein, gene A1BG, score 300). Representative MS/MS spectrums of particular peptides of C9 protein are shown. The red peaks indicate matched b-ion and y-ion series. The sequences of precursor ions, ([M + 2H]2+, a:733.296, b:1395.453, c:728.271), were analyzed by MS/MS to be DVVLTTTFVDDIK (a), GTVIDVTDFVNWASSINDAPVLISQK (b), and AIEDYINEFSVR (c). (PDF) [file pone.0198472.s002.pdf]
