# Supplementary material for: Immunoproteomic identification of anti-C9 autoimmune antibody in patients with seronegative obstetric antiphospholipid syndrome
Source: PLoS One. 2018 Jun 12;13(6):e0198472. doi: 10.1371/journal.pone.0198472 (PMC5997311; doi:10.1371/journal.pone.0198472)
Supplement: S1 Table — The Mascot search showed that only the complement molecule C9 had high score, whereas the other candidate proteins did not match the molecular weight according to their positions in 2-DE analysis. (PDF) [file pone.0198472.s003.pdf]

S1 Table

MATRIX SCIENCE MASCOT Search Results

User :
E-mail :
Search title : Submitted from Untitled by Mascot Daemon on 98S0322
MS data file : D:\Katayama\GY4520N1__01_991.d\ProteinAnalysisResults.mgf
Database : SwissProt 2018_02 (556,825 sequences; 199,652,254 residues)
Taxonomy : Homo sapiens (human) (20,317 sequences)
Timestamp : 30 Mar 2018 at 12:30:15 GMT

Re-search ◉ All ○ Non-significant ○ Unassigned [help] Export As XML

Not what you expected? Try the select summary.

Search parameters

Type of search : MS/MS Ion Search
Enzyme : Trypsin
Fixed modifications : Carbamidomethyl (C)
Variable modifications : Deamidated (NQ), Oxidation (M)
Mass values : Monoisotopic
Protein mass : Unrestricted
Peptide mass tolerance : ± 0.5 Da
Fragment mass tolerance : ± 0.8 Da
Max missed cleavages : 2
Instrument type : ESI-TRAP
Number of queries : 4,125

Score distribution
Modification statistics
Legend

Protein Family Summary

Format | Significance threshold p< 0.05 | Max. number of families AUTO | [help]
Display non-sig. matches ☐ | Dendrograms cut at 0
Preferred taxonomy .... Homo sapiens (human)

Sensitivity

Proteins (30) | Report Builder | Unassigned (3928) | § permalink

Protein hits (31 proteins)

Columns: Standard (12 out of 16)

Filters: (none)

Export as CSV

Family	M	DB	Accession	↓Score	Mass	Matches	Match (sig)	Sequences	Seq (sig)	emPAI	Description
1	1	SwissProt	CO9_HUMAN	2897	64615	122	122	22	22	3.30	Complement component C9 OS=Homo sapiens OX=9606 GN=C9 PE=1 SV=2
2	1	SwissProt	A1BG_HUMAN	301	54790	11	11	10	10	1.01	Alpha-1B-glycoprotein OS=Homo sapiens OX=9606 GN=A1BG PE=1 SV=4
3	1	SwissProt	IGHA1_HUMAN	265	38486	9	9	6	6	0.88	Immunoglobulin heavy constant alpha 1 OS=Homo sapiens OX=9606 GN=IGHA1 PE=1 SV=2
4	1	SwissProt	AFAM_HUMAN	220	70963	12	12	10	10	0.81	Afamin OS=Homo sapiens OX=9606 GN=AFM PE=1 SV=1
5	1	SwissProt	ANT3_HUMAN	192	53025	6	6	6	6	0.48	Antithrombin-III OS=Homo sapiens OX=9606 GN=SERPINC1 PE=1 SV=1
6	1	SwissProt	A1AT_HUMAN	133	46878	2	2	2	2	0.16	Alpha-1-antitrypsin OS=Homo sapiens OX=9606 GN=SERPINA1 PE=1 SV=3
3	2	SwissProt	IGA2_HUMAN	123	49817	5	5	4	4	0.32	Immunoglobulin alpha-2 heavy chain OS=Homo sapiens OX=9606 PE=1 SV=1
7	1	SwissProt	CO5_HUMAN	105	189897	2	2	2	2	0.04	Complement C5 OS=Homo sapiens OX=9606 GN=C5 PE=1 SV=4
8	1	SwissProt	VTDB_HUMAN	105	54526	3	3	3	3	0.21	Vitamin D-binding protein OS=Homo sapiens OX=9606 GN=GC PE=1 SV=1
9	1	SwissProt	LUM_HUMAN	95	38747	2	2	1	1	0.09	Lumican OS=Homo sapiens OX=9606 GN=LUM PE=1 SV=2
10	1	SwissProt	K2C1_HUMAN	87	66170	2	2	2	2	0.11	Keratin, type II cytoskeletal 1 OS=Homo sapiens OX=9606 GN=KRT1 PE=1 SV=6
11	1	SwissProt	ANGT_HUMAN	85	53406	3	3	3	3	0.22	Angiotensinogen OS=Homo sapiens OX=9606 GN=AGT PE=1 SV=1
12	1	SwissProt	HEMO_HUMAN	83	52385	2	2	2	2	0.14	Hemopexin OS=Homo sapiens OX=9606 GN=HPX PE=1 SV=2
13	1	SwissProt	A2AP_HUMAN	74	54873	3	3	3	3	0.21	Alpha-2-antiplasmin OS=Homo sapiens OX=9606 GN=SERPINF2 PE=1 SV=3
14	1	SwissProt	TRY1_HUMAN	69	27111	1	1	1	1	0.14	Trypsin-1 OS=Homo sapiens OX=9606 GN=PRSS1 PE=1 SV=1
15	1	SwissProt	CO4A_HUMAN	68	194261	1	1	1	1	0.02	Complement C4-A OS=Homo sapiens OX=9606 GN=C4A PE=1 SV=2
16	1	SwissProt	APOA1_HUMAN	63	30759	1	1	1	1	0.12	Apolipoprotein A-I OS=Homo sapiens OX=9606 GN=APOA1 PE=1 SV=1
17	1	SwissProt	CERU_HUMAN	50	122983	1	1	1	1	0.03	Ceruloplasmin OS=Homo sapiens OX=9606 GN=CP PE=1 SV=1
18	1	SwissProt	TRY6_HUMAN	46	27090	1	1	1	1	0.14	Putative trypsin-6 OS=Homo sapiens OX=9606 GN=PRSS3P2 PE=5 SV=2
19	1	SwissProt	KNG1_HUMAN	45	72996	1	1	1	1	0.05	Kininogen-1 OS=Homo sapiens OX=9606 GN=KNG1 PE=1 SV=2
20	1	SwissProt	KCNC1_HUMAN	42	58475	1	1	1	1	0.06	Potassium voltage-gated channel subfamily C member 1 OS=Homo sapiens OX=9606 GN=KCNC1 PE=1 SV=1
21	1	SwissProt	FIBG_HUMAN	37	52106	1	1	1	1	0.07	Fibrinogen gamma chain OS=Homo sapiens OX=9606 GN=FGG PE=1 SV=3
22	1	SwissProt	HXK2_HUMAN	37	103739	1	1	1	1	0.03	Hexokinase-2 OS=Homo sapiens OX=9606 GN=HK2 PE=1 SV=2
23	1	SwissProt	CO3_HUMAN	37	188569	1	1	1	1	0.02	Complement C3 OS=Homo sapiens OX=9606 GN=C3 PE=1 SV=2
24	1	SwissProt	LYZL4_HUMAN	33	16878	1	1	1	1	0.22	Lysozyme-like protein 4 OS=Homo sapiens OX=9606 GN=LYZL4 PE=2 SV=1
25	1	SwissProt	IF2GL_HUMAN	30	51766	1	1	1	1	0.07	Putative eukaryotic translation initiation factor 2 subunit 3-like protein OS=Homo sapiens OX=9606 GN=EIF2S3L PE=5 SV=2
26	1	SwissProt	ZMYM3_HUMAN	30	156101	1	1	1	1	0.02	Zinc finger MYM-type protein 3 OS=Homo sapiens OX=9606 GN=ZMYM3 PE=1 SV=2
27	1	SwissProt	NFKB2_HUMAN	28	97373	1	1	1	1	0.04	Nuclear factor NF-kappa-B p100 subunit OS=Homo sapiens OX=9606 GN=NFKB2 PE=1 SV=4
28	1	SwissProt	EGFR_HUMAN	27	137612	1	1	1	1	0.03	Epidermal growth factor receptor OS=Homo sapiens OX=9606 GN=EGFR PE=1 SV=2
29	1	SwissProt	APOA4_HUMAN	26	45371	1	1	1	1	0.08	Apolipoprotein A-IV OS=Homo sapiens OX=9606 GN=APOA4 PE=1 SV=3
30	1	SwissProt	PROM2_HUMAN	25	93079	1	1	1	1	0.04	Prominin-2 OS=Homo sapiens OX=9606 GN=PROM2 PE=1 SV=1
